# CircRNA hsa_circ_0110102 inhibited macrophage activation and hepatocellular carcinoma progression via miR-580-5p/PPARα/CCL2 pathway

**DOI:** 10.18632/aging.202900

**Published:** 2021-04-23

**Authors:** Xinxing Wang, Wei Sheng, Tao Xu, Jiawen Xu, Ruyi Gao, Zhenhai Zhang

**Affiliations:** 1 Department of Hepatobiliary Surgery, Shandong Provincial Hospital Affiliated to Shandong First Medical University, Jinan 250021, Shandong, China; 2 Department of Oncology, Shandong Provincial Hospital Affiliated to Shandong First Medical University, Jinan 250021, Shandong, China; 3 Department of Gastrointestinal Surgery, Shandong Provincial Hospital Affiliated to Shandong First Medical University, Jinan 250021, Shandong, China; 4 Department of Pathology, Shandong Provincial Hospital Affiliated to Shandong First Medical University, Jinan 250021, Shandong, China

**Keywords:** hepatocellular carcinoma, hsa_circ_0110102, miR-580-5p, CCL2, cyclooxygenase-2

## Abstract

Circular RNAs (circRNAs) have critical regulatory roles in tumor biology. However, their contributions in hepatocellular carcinoma (HCC) still remain enigmatic. The present study aimed to investigate the molecular mechanisms underlying the involvement of hsa_circ_0110102 in the occurrence and development of HCC. The expression level of hsa_circ_0110102 was significantly downregulated in HCC cell lines and tissues, which was associated with poor prognosis. Knockdown hsa_circ_0110102 significantly promoted cell proliferation, migration, and invasion. Moreover, the interaction between hsa_circ_0110102 and miR-580-5p was predicted and verified by luciferase assay and RNA pull-down. The findings indicated that hsa_circ_0110102 functioned as a sponge for miR-580-5p. Moreover, miR-580-5p directly bound to the 3′ UTR of PPARα, which decreased the production and release of C-C chemokine ligand 2 (CCL2) in HCC cells. CCL2 could activate the cyclooxygenase-2/prostaglandin E2 (COX-2/PGE2) pathway in macrophage via FoxO1 in a p38 MAPK–dependent manner. Furthermore, the Δ256 mutant of FoxO1 showed no activation effect. These results concluded that hsa_circ_0110102 acted as a sponge for miR-580-5p and inhibited CCL2 secretion into tumor microenvironment by decrease the expression of PPARα in HCC cells, then inhibited the pro-inflammatory cytokine release from macrophages by regulating the COX-2/PGE2 pathway. In conclusion, hsa_circ_0110102 served as a potential prognostic predictor or therapeutic target for HCC.

## INTRODUCTION

Hepatocellular carcinoma (HCC) is the most frequent primary malignancy of the liver and the third leading cause of cancer deaths worldwide, accounting for about 90% of all cases of primary liver cancers [1]. China accounts for more than 50% of the total HCC cases worldwide. The main risk factors for HCC are chronic infection with hepatitis B virus (HBV) and hepatitis C virus and nonalcoholic fatty liver disease. Great efforts have been made to take diagnosis and treatment methods to new heights in the last decades, but the mortality rate is still unsatisfactory. Therefore, determining the risk factors of HCC occurrence and revealing the molecular mechanism of its promotion are urgently needed.

Circular RNAs (circRNAs) are a subclass of transcripts with covalently closed-loop structures that lack classic 5′ caps or 3′ polyadenylated tails, which used to be regarded as by-products of erroneous splicing [2]. However, a large number of recent studies showed that circRNAs were important and served various functions in the nervous system, carcinogenesis, cardiovascular diseases, and immunomodulatory and metabolic disorders [3, 4]. CircRNAs regulate target genes or protein expression in various manners, for example, by functioning as microRNA (miRNA) sponges to show miRNA inhibitory effect via competing endogenous RNA (ceRNA) [5], by interfering with their parental genes via modulating alternative splicing or transcription [6], and by interacting with RNA-binding proteins (RBPs) as scaffolds for the assembly of protein complexes [7]. Recently, numerous circRNAs have been found to be dysregulated in HCC. For example, circBACH1 (hsa_circ_0061395) is significantly upregulated in HCC tissue, and circBACH1 knockdown suppresses the proliferation and increases the apoptosis of HCC cells by regulating p27 repression via HuR [8]. CircASAP1 acts as a ceRNA for miR-326 and miR-532-5p to mediate tumor-associated macrophage (TAM) infiltration [9]. As a consequence, emerging evidence supported the critical roles of circRNA in HCC tumorigenesis and development.

C-C chemokine ligand 2 (CCL2) is overexpressed in HCC and regulated by the activation of PPARα [10]. In the tumor microenvironment, the interaction between CCL2 and C-C motif chemokine receptor 2 (CCR2) regulates the chemotaxis of TAMs and contributes to cancer progression. Cyclooxygenase-2 (COX-2), as a key enzyme in the synthesis of prostaglandin E2 (PGE2), is an important inflammatory factor and overexpressed in many metastatic tumors [11]. COX-2 is crucial in angiogenesis, apoptosis, inflammation, and metastasis [12], and is proposed to be a potential tumor therapeutic target [13]. Nonsteroidal anti-inflammatory drugs, the main available COX-2 inhibitors, are widely used in cancer treatment [14].

In this study, a novel circRNA hsa_circ_0110102 was identified and its biological roles in the HCC progression were investigated. hsa_circ_0110102 targeted the miR-580-5p/PPARα/CCL2 in HCC. Moreover, CCL2 secreted into the tumor microenvironment induced the expression of COX-2 and the release of PGE2 in a FoxO1-dependent manner in macrophages, and then promoted the proliferation of HCC cells. Overall, these findings indicated that hsa_circ_0110102 might act as a novel biomarker for HCC prognosis and as a promising therapeutic target.

## MATERIALS AND METHODS

### Reagents

Recombinant human CCL2 for cell treatments was obtained from R&D (MN, USA; #279-MC-050). AS1842856 (#344355) and phorbol 12-myristate 13-acetate (PMA, #P8139) were obtained from Sigma–Aldrich (MO, USA). WY-14643 (#S8029), GW4671 (#S2798), SB203580 (#S1076), SP600125 (#S1460), and SCH772984 (S7101) were obtained from Selleck (Shanghai, China). The Dual-Luciferase Reporter Assay System (E1910) was obtained from Promega (WI, USA). The SimpleChIP (chromatin immunoprecipitation) Plus Sonication Chromatin IP Kit (#56383) and Alexa Fluor 488–conjugated CD68 antibody (#24850) were purchased from Cell Signaling Technology (MA, USA). The Alexa Fluor 647–conjugated CCR2 antibody (#ab225432) was obtained from Abcam (Cambridge, UK). The Lipofectamine 2000 (#11668019), negative control (miR-NC), miR-580-5p mimic (#4464066), and inhibitor (AM17000) were obtained from Thermo Fisher Scientific (Runcorn, Cheshire, UK). siRNA- Zinc Finger Protein 562 (ZNF562), siRNA-circ-102231, and the negative control were purchased from GenePharma Biotechnology (Shanghai, China). The siRNA target site of hsa_circ_0110102 was 5′-ACAGTGGAGAAAGGTAAATGCAA-3′, and that of ZNF562 was 5′-GTCATTGATAACATCTTATCAGG-3′. The construction of hsa_circ_0110102 overexpression in the Ubi-MCS-Luc-IRES-Puromycin vector was performed by Biosyntech Co., Ltd (Suzhou, China).

### Clinical tissue samples and cell culture

HCC tissues used in this study involved 17 patients of both sexes with previously diagnosed HCC (13 men and 4 women) and an average age of 57 ± 12 years at Shandong Provincial Hospital Affiliated to Shandong First Medical University, Jinan, China. Tumor tissues and adjacent tissue samples were immediately collected, placed in liquid nitrogen, and stored at −80°C until further processing. All samples used in this study were approved by the Committees for the Ethical Review of Research Involving Human Subjects at Shandong Provincial Hospital.

Human HCC cell lines HepG2, MHCC-97H, Huh-7, Hep-3B, and SMCC-7721; human normal liver cell line (LO2); and human monocytic leukemia cell THP-1 were purchased from the Type Culture Collection of the Chinese Academy of Sciences (Shanghai, China). All these cell lines were cultured in Dulbecco’s modified Eagle’s medium or Roswell Park Memorial Institute (RPMI) 1640 medium supplemented with 10% fetal bovine serum (Gibco) at 37°C.

The THP-1 and Huh-7 co-culture system was established to simulate the microenvironment of HCC as described in a previous study [15]. Briefly, THP-1 cells were seeded into six-well plates and differentiated with 200 ng/mL PMA for 24 h. Moreover, Huh-7 cells were transfected with an expression vector for 24 h and then seeded into a Transwell insert at a density of 1.5 × 10^5^ cells per chamber. The differentiated THP-1 cells were washed three times with phosphate-buffered saline (PBS) and then co-cultured with Huh-7 cells for another 24 h. At the end of the treatment, THP-1 cells were harvested for flow cytometry (FCM), quantitative reverse transcription–polymerase chain reaction (RT-qPCR), and Western blot analysis, and the medium was collected for enzyme-linked immunosorbent assay (ELISA) assay.

### RNA immunoprecipitation

RNA immunoprecipitation (RIP) was conducted using a Magna RIP RBP immunoprecipitation kit (Millipore, CA, USA) following the manufacturer’s protocol. Briefly, anti-AGO2 antibody and rabbit immunoglobulin (IgG) were incubated with magnetic beads at room temperature for 30 min to generate antibody-coated beads. Approximately 1 × 10^7^ cells were lysed and mixed at 4°C overnight. After washing, co-immunoprecipitated RNA was extracted and detected using RT-qPCR.

### Pull-down assay

Biotin-labeled hsa_circ_ 0110102 or oligo probes (GenePharma) were pre-incubated with Streptavidin-Dyna beads M-280 (#11206D, Invitrogen, Carlsbad, CA, USA). hsa_circ_0110102-overexpressing and control cells were lysed and incubated with the beads at 4°C overnight. Then, RNA was extracted and measured by RT-qPCR.

### Fluorescence *in situ* hybridization

Huh-7 cells were seeded into confocal dishes and fixed with 4% paraformaldehyde overnight. A Cy3-labeled hsa_circ_0110102 probe (5′-GGTGCAATCGGACAC CTTGGATATTGCAGACA-3′-Cy3) was designed and synthesized by GenePharma. Nuclei were counterstained with 4,6-diamidino-2-phenylindole (DAPI). All processes were conducted following the manufacturer’s protocols. Images were obtained using an FV3000 microscope (Olympus, Japan).

### Western blot analysis

The proteins in the cytoplasm and nucleus were extracted using a kit from Beyotime (Shanghai, China). They were then separated by SDS-PAGE, transfected onto a PVDF membrane, immunoblotted with indicated primary antibodies and HRP-linked antibodies (1:5000 dilution; anti-rabbit IgG, #7074; anti-mouse IgG, #7076; CST), and visualized using a Tanon-5200 Chemiluminescence Imager (Tanon, Shanghai, China) with ECL substrate (Millipore). Primary antibodies used were as follows: CCL2 (1:1000 dilution; ab9669, Abcam), COX-2 (1:1000 dilution; ab15191, Abcam), FoxO1 (1:1000 dilution; ab39670, Abcam), PPARα (1:1000 dilution; ab215270, Abcam) and β-actin (1:5000 dilution; #3700, CST).

### Cell proliferation assay

A cell counting kit-8 (CCK-8, #HY-K0301, MCE, Shanghai, China) was used to assess the proliferation of cells. Transfected cells were seeded into 96-well plates at a density of 5 × 10^3^ cells per well and incubated at 37°C for the indicated time. Then, 10 μL of CCK-8 solution was added to each well and incubated for 2 h before detection, and then absorbance at 450 nm was measured using a microplate reader (Varioskan LUX, Thermo Fisher, USA).

For the EdU staining assay, an EdU staining kit (iFluor 647, ab222421, Abcam) was used following the manufacturer’s protocol. In brief, the cells were seeded into six-well plates and transfected for 48 h. Then, the cells were stained with EdU solution for 2 h, fixed with 4% paraformaldehyde, and cultured with 0.5% Triton X-100 for 10 min. Finally, the cells were stained with DAPI for 10 min. The images were collected using an FV3000 microscope.

### Quantitative reverse transcription–polymerase chain reaction

Total RNA was extracted using a TRIzol reagent (#15596-026, Invitrogen), and nuclear and cytoplasmic RNA was extracted using the PARIS Kit (#AM1921, Thermo Fisher). RT-qPCR kits were purchased from TaKaRa Bio Inc (Dalian, China), and cDNA was synthesized following the manufacturer’s protocol. The QuantStudio 5 Real-Time PCR System (Applied Biosystems, Waltham, MA, USA) and the SYBR Green (no. B21202, Bimake, TX, USA) were used for the RT-qPCR assay. The expression levels of mRNA were normalized to GAPDH mRNA. Primers were purchased from Genscript Biotech Co., Ltd. (Nanjing, China). For the miRNA assay, miRNA-specific Taqman PCR primers were obtained from Life Technologies (CA, USA). The gene levels were normalized to U6 or GAPDH to generate a 2^−ΔΔC^^t^ value for the relative expression of each transcript. Primer sequences used in the experiments were as follows:

**Table:** 

**Gene**	**Forward (5′-3′)**	**Reverse (5′-3′)**
hsa_circ_0110102	CCCAGGGAACCAATCTGTCC	GGTGAACTCCACAGCCACAT
ZNF562	CCCAGGGAACCAATCTGTCC	GGTGAACTCCACAGCCACAT
CCL2	GCTCATAGCAGCCACCTCATTC	CCGCCAAAATAACCGATGTGATAC
COX-2	CTGGCGCTCAGCCATACAG	CGCACTTATACTGGTCAAATCCC
GAPDH	GGAGCGAGATCCCTCCAAAAT	GGCTGTTGTCATACTTCTCATGG

### Flow cytometry

Huh-7 cells were transfected with miR-580-5p mimic or hsa_circ_0110102, and then co-cultured with THP-1 cells for 24 h. THP-1 was incubated with CD68 and CCR2 antibodies for 1 h at 4°C. then washed with PBS for three times, finally resuspended in 200 μL of PBS, and analyzed by flow cytometry.

For cell cycle analysis, HepG2 and Huh-7 cells were trypsinized and fixed with 70% ethanol overnight at 4°C, followed by staining with a propidium iodide (PI) reagent. The percentages of cells in the G0/G1, S, and G2/M phases were recorded using flow cytometry (BD Biosciences, NY, USA). The results obtained were analyzed using FlowJo software.

### ELISA

The THP-1 cells were treated with 50 ng/mL rCCL2 or transfected with the expression vector, and then treated with or without 1μM AS1842856 or infected with sh-FoxO1 for 24 h. The culture supernatant was collected and used to detect the levels of vascular endothelial growth factor (VEGF; cat no. DVE00), COX-2 (cat no. AF4198), IL-6 (cat no. D6050), and PGE2 (cat no. KGE004B) secreted using ELISA kits (all from R&D). The absorbance was measured at 450 nm using a microplate reader.

### *In vitro* migration and invasion assays

Cell migration and invasion were measured using Transwell assay as described in a previous study [16].

### Plasmid constructs and transfection

pSELECT-HA-mFoxO1-wild-type (Addgene plasmid # 83308) and Δ256 (Addgene plasmid # 83379) were gifts from Steven Abcouwer [17]. The full-length CCL2 cDNA was amplified and cloned into a pcDNA3.1 expression vector. The CCL2 promoter sequence from positions −500 to +1 and COX-2 promoter sequence from positions −1122 to +27 relative to the transcriptional start site were subcloned into a pGL3-basic vector; the point mutation in the FoxO1-binding site of COX-2 promoter was generated by site-directed mutagenesis, which was spliced by overlap extension. FoxO1 shRNA and scrambled shRNA were purchased from Obio Technology Co., Ltd. (Shanghai, China). The cells were seeded into six-well plates for overnight culture and then transfected with hsa_circ_0110102 overexpression vector or siRNA, miR-NC, miR-580-5p mimic, or inhibitor with Lipofectamine 2000 for 48 h; blank vector-transfected cells were used as controls.

### ChIP assay

The *in vivo* association of FoxO1 with human COX-2 promoter was detected by performing ChIP analysis as described in a previous study using a SimpleChIP Enzymatic Chromatin IP Kit (#9002, CST, USA) following the manufacturer’s protocol. After purification of DNA, the DNA fragment size was determined by electrophoresis on a 1% agarose gel. The ChIP-PCR was used for validation with the forward primer 5′-CACCGGGCTTACGCAATTTT-3′ and the reverse primer 5′-ACGCTCACTGCAAGTCGTAT-3′, which were specifically designed from the COX-2 promoter region (139 to +29).

### Luciferase assays

The cells were transfected with luciferase reporter plasmids and subsequently incubated for 24 h in a complete medium. The luciferase activity was measured using the Dual-Luciferase Reporter Assay System (Promega) and normalized to *Renilla* luciferase values. Measurements for three biological replicates were taken and averaged.

### Statistical analysis

Data were expressed as mean ± standard error of mean. SPSS 21.0 was used for statistical analysis. The unpaired Student *t* test was used for comparision between the two groups. Two-way analysis of variance analysis using the Tukey test was employed to compare three groups or more. A *P* value <0.05 was considered statistically significant.

## RESULTS

### hsa_circ_0110102 was downregulated in HCC cell lines and tissues

**Figure 1:**
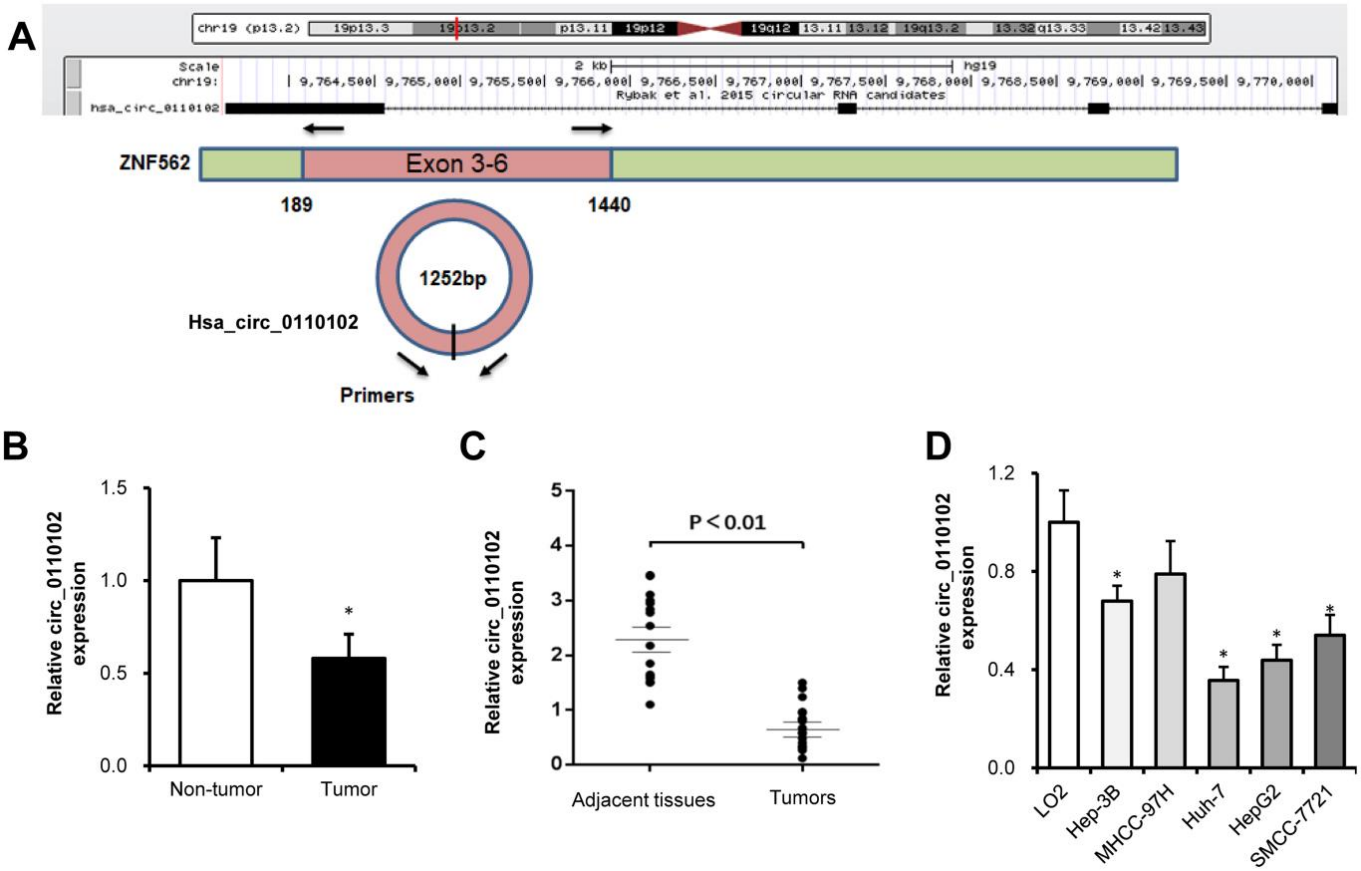
**hsa_circ_0110102 was downregulated in HCC tumor tissues and cell lines.** (**A**) The genomic loci of hsa_circ_0110102 and the schematic model of the primers of hsa_circ_0110102. The primers targeted the backsplice junction of hsa_circ_0110102. (**B**) The relative expression of hsa_circ_0110102 was validated in five pairs of normal and HCC tissues from the GSE135806 dataset. (**C**) The expression of hsa_circ_0110102 mRNA was analyzed in 17 tumor tissues and 17 nontumor tissues by RT-qPCR. (**D**) The expression levels of hsa_circ_0110102 in five HCC cell lines and LO2 cell were examined by RT-PCR. Data are presented as mean ± standard error. ^*^, *P* < 0.05.

hsa_circ_0110102, with the 1252-bp spliced mature sequence length, was located at chr19:9763628-9770143 and derived from exons 3 to 6 of the *ZNF562* gene (Figure 1A). The published GSE135806 dataset was used to identify circRNAs differentially expressed between five HCC and five adjacent normal tissues from HBV-related male patients with HCC, aged 43–57 years [18]. Compared with control tissues, the expression of hsa_circ_0110102 was found to be downregulated in HCC tissues (Figure 1B). Compared with matched normal control tissues, hsa_circ_0110102 had a low expression in 17 HCC tissues collected from the Shandong Provincial Hospital (Figure 1C). In the HCC cell lines (Hep3B, MHCC-97H, Huh-7, HepG2, and SMCC-7721), the expression of hsa_circ_0110102 was significantly lower than that in human normal LO2 hepatocytes (Figure 1D). As Huh-7 and HepG2 cells showed lower hsa_circ_0110102 levels than those in other HCC cells, these two cell lines were used in the subsequent studies to reveal the tumor-suppressive effect in HCC.

### hsa_circ_0110102 knockdown promoted HCC cell growth, migration, and invasiveness

**Figure 2:**
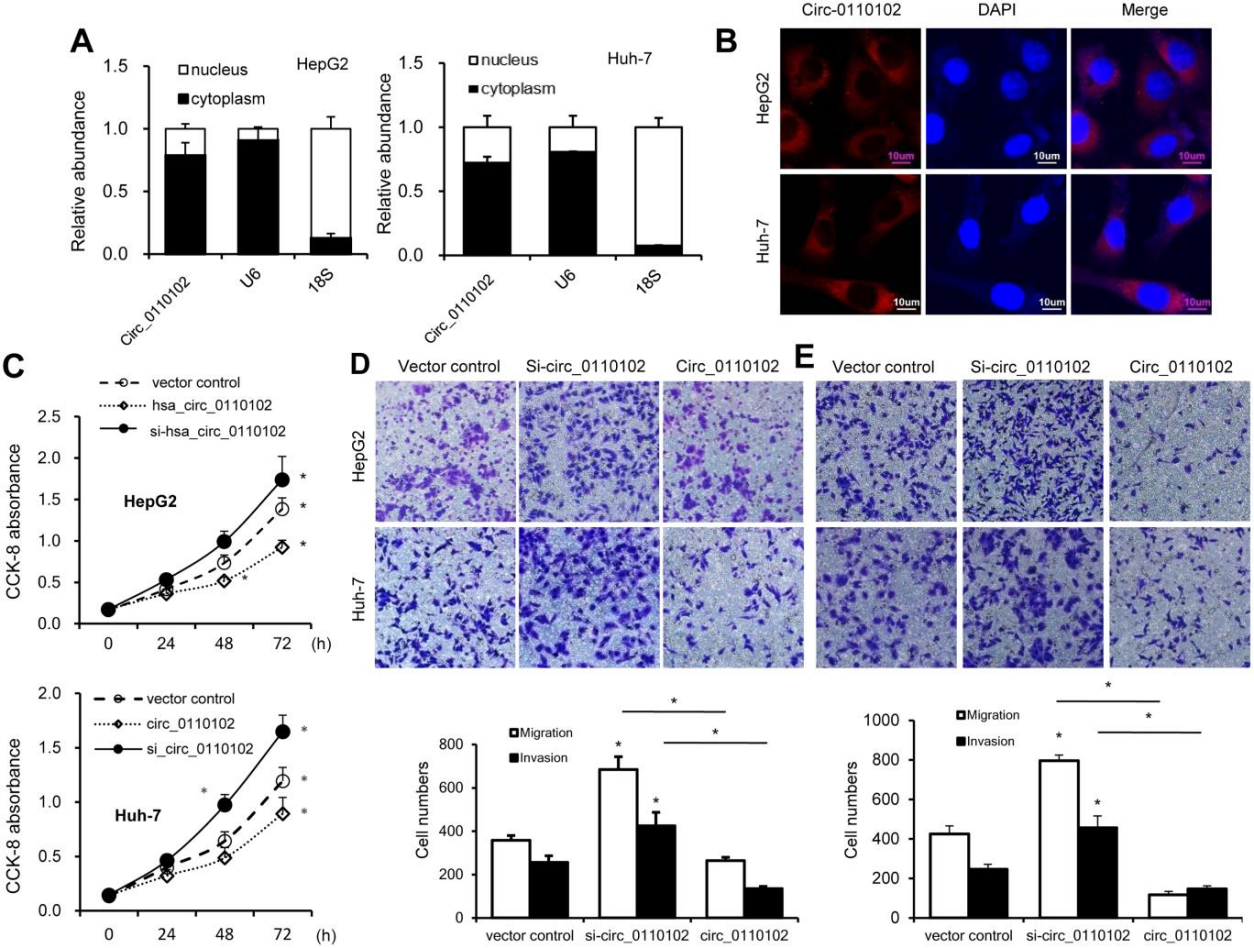
**hsa_circ_0110102 inhibited the proliferation and migration ability of HCC *in vitro*.** (**A**) The relative quantity of hsa_circ_0110102, U6, and 18S in the cytoplasmic and nuclear fractions of HCC cells was determined by RT-qPCR. (**B**) Subcellular localization of hsa_circ_0110102 was determined by the FISH assay. (**C**) CCK-8 assays were performed to assess the proliferation of Huh-7 and HepG2 cells after transfection. (**D** and **E**) The ability of cell migration and invasion was measured by Transwell assay in HCC tumor cell lines HepG2 and Huh-7 with si-circ_0110102 and circ_0110102 transfection. The representative magnified sections of Transwell cell staining images are shown. Statistical results based on three independent experiments are shown in the right (200×). Data are presented as mean ± standard error. ^*^, *P* < 0.05.

Next, the relative abundance of hsa_circ_0110102 in the nucleus and cytoplasm of Huh-7 and HepG2 cells was examined via nuclear mass separation assays (Figure 2A) and fluorescence *in situ* hybridization (FISH; Figure 2B), indicating that hsa_circ_0110102 was mainly located in the cytoplasm.

hsa_circ_0110102-specific small interfering RNAs (siRNAs) were used to downregulate the expression levels of hsa_circ_0110102 to analyze the role of hsa_circ_0110102 in HCC. Thus, siRNAs targeting the backsplice junction sequence of hsa_circ_0110102 and the full-length ZNF562 were designed. siRNA targeting the backsplice junction knocked down only the circular transcript and did not affect the expression of linear species. On the contrary, siRNA targeting linear transcript knocked down only the ZNF562 linear transcript but not the circular transcript (Supplementary Figure 1). Huh-7 and HepG2 cells were transfected with hsa_circ_0110102 expression vector and siRNA, followed by CCK-8 assay and migration and invasion assay. hsa_circ_0110102 knockdown was found to dramatically induce proliferation, cell migration, and invasiveness, which were inhibited by the overexpression of hsa_circ_0110102 (Figure 2C–2E).

### hsa_circ_0110102 acted as a sponge for miR-580-5p

**Figure 3:**
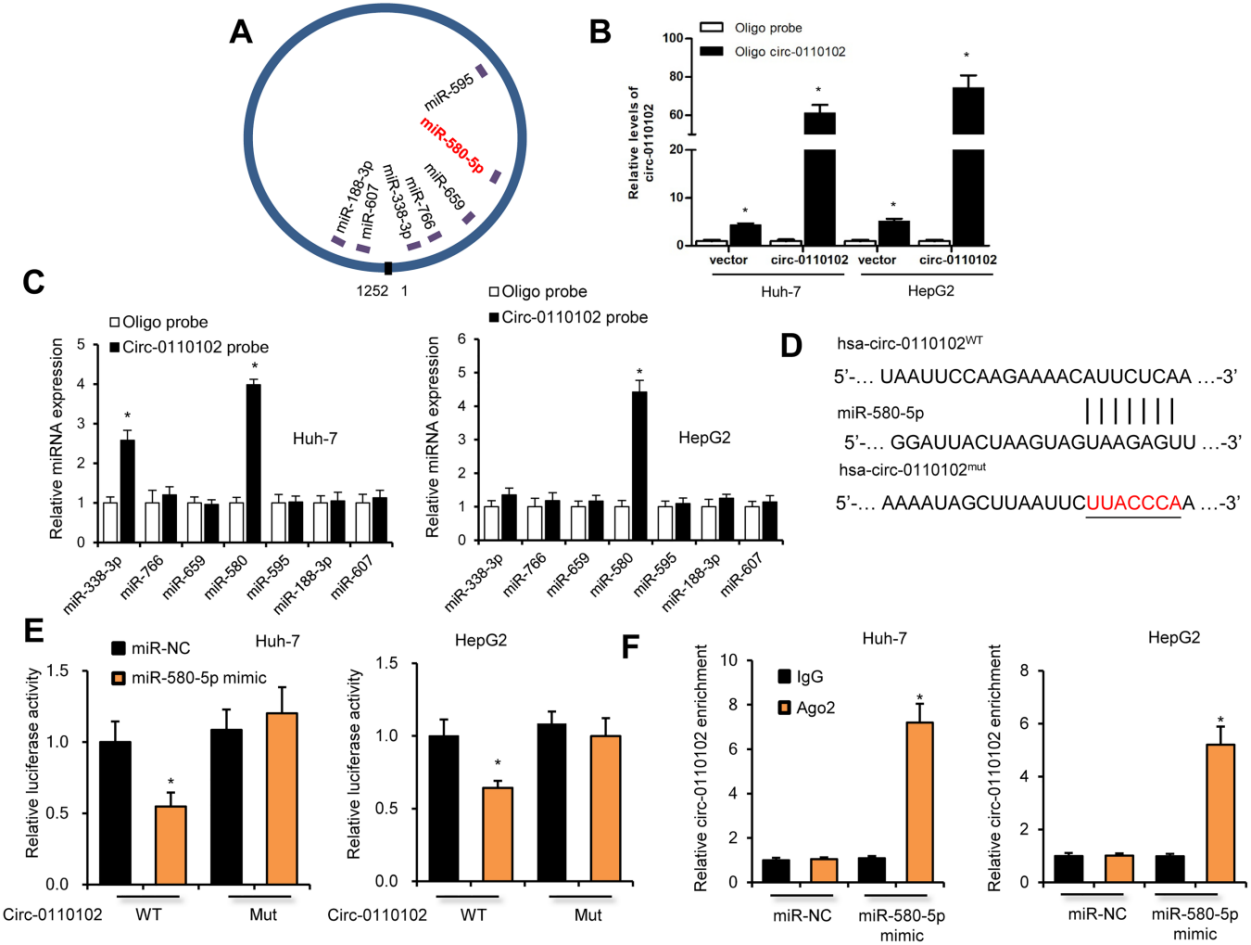
**hsa_circ_0110102 served as a sponge for miR-580-5p.** (**A**) Schematic illustration of the predicted binding sites between hsa_circ_0110102 and seven candidate miRNAs. (**B**) RNA pull-down assay with hsa_circ_0110102 or oligo probes. (**C**) Relative levels of seven miRNAs in cell lysate pulled down by oligo or hsa_circ_0110102 probes were detected by RT-qPCR. (**D**) The predicted binding sites of miR-580-5p on hsa_circ_0110102. (**E**) Relative luciferase activity in Huh-7 and HepG2 cells after transfection of hsa_circ_0110102 WT/Mut and 3′-UTR of miR-580-5p. (**F**) Anti-AGO2 RIP was performed in Huh-7 and HepG2 cells after miR-580-5p NC or mimic transfection. Data are presented as mean ± standard error. ^*^, *P* < 0.05.

The target miRNAs of hsa_circ_0110102 were predicted by the https://circinteractome.nia.nih.gov/. Seven candidate miRNAs (miR-338-3p, miR-766, miR-659, miR-580-5p, miR-595, miR-188-3p, and miR-607) were chosen for the next study according to their higher context score percentile (Supplementary Figures 2 and 3A). The biotin-labeled hsa_circ_0110102 probe robustly enriched hsa_circ_0110102 compared with the oligo probe in HCC cells, verifying the efficiency of the pull-down assay (Figure 3B). RNA extracted from the pull-down assay was analyzed using RT-qPCR. The hsa_circ_0110102 probe was found to enrich mostly miR-580-5p compared with other miRNAs in both Huh-7 and HepG2 cells (Figure 3C). The following luciferase reporter assay results showed that miR-580-5p mimics remarkably reduced the luciferase activity of hsa_circ_0110102, while miR-580-5p overexpression showed no effect on the activity of LUC-circ_0110102-mutant reporter gene (Figure 3D and 3E). Next, the anti-AGO2 RIP assay found that miR-580-5p mimics pulled down hsa_circ_0110102 through the anti-AGO2 antibody, and not control IgG (Figure 3F). Overall, the results demonstrated that hsa_circ_0110102 functioned as a sponge for miR-580-5p.

### miR-580-5p in HCC was associated with poor prognosis

**Figure 4:**
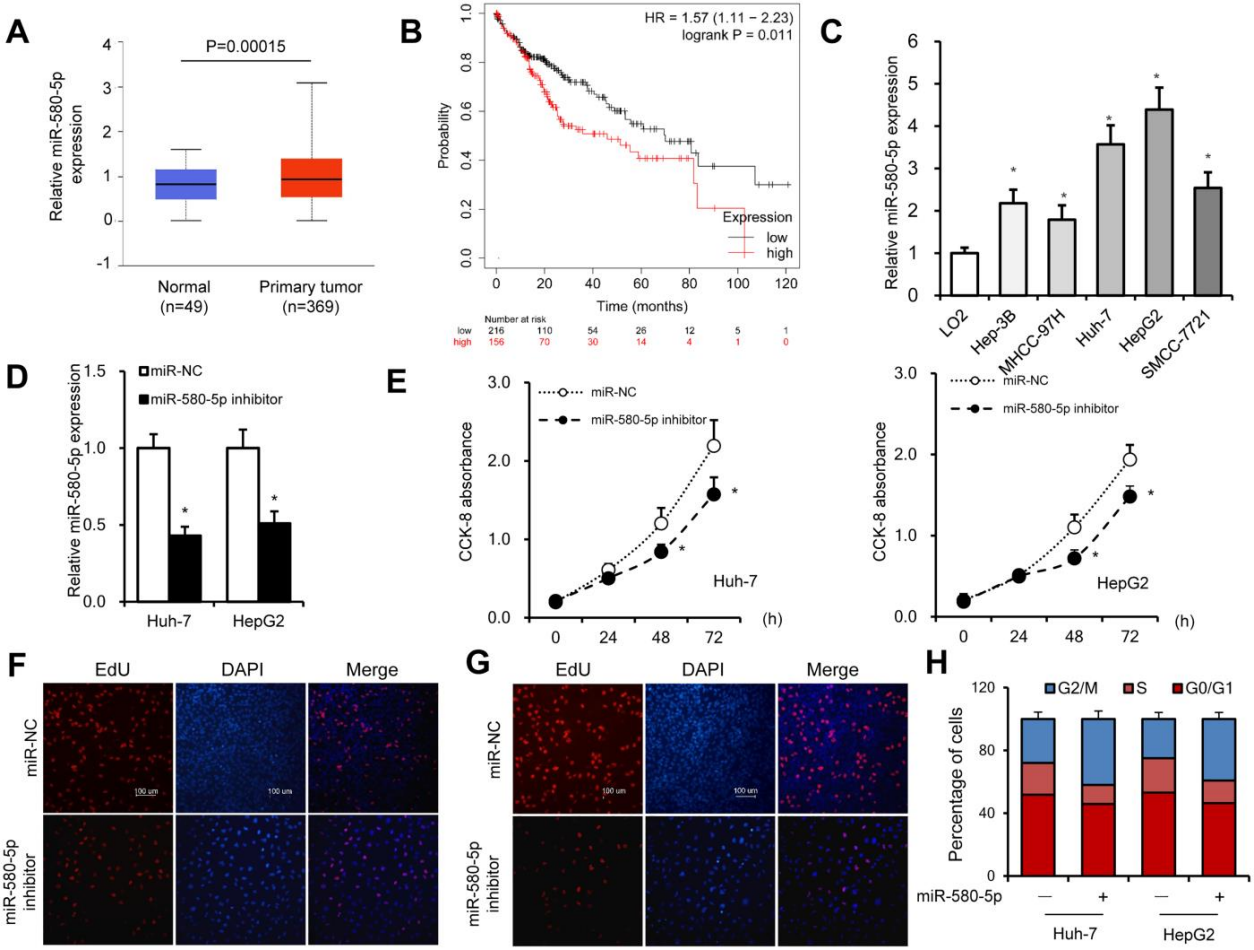
**miR-580-5p was overexpressed in HCC tumor tissues and cell lines.** (**A**) miR-580-5p levels in 49 normal and 369 primary HCC tissues. (**B**) Kaplan–Meier survival curve of patients in low- and high-expression miR-580-5p groups. (**C**) miR-580-5p levels in five HCC cell lines and LO2 cells were examined by RT-PCR. Huh-7 and HepG2 cells were transfected with miR-580-5p NC or mimic for 48 h. (**D**) The miR-580-5p levels were examined with RT-qPCR. (**E**) Cell viability was detected using the CCK-8 assay. (**F** and **G**) EdU assays were used to detect the DNA synthesis in Huh-7 and HepG2 cells. (**H**) Cell cycle was detected using flow cytometry assays. Data are presented as mean ± standard error. ^*^, *P* < 0.05.

An increase in the expression of miR-580-5p in HCC tissues was found by analyzing the The Cancer Genome Atlas (TCGA) database (http://ualcan.path.uab.edu/analysis-mir.html) (Figure 4A). The KM-plotter (http://kmplot.com/analysis/) database also showed that miR-580-5p was associated with a lower survival rate in 372 patients with HCC (Figure 4B). The higher expression levels of miR-580-5p were observed in five HCC cell lines (Figure 4C).

Next, the effect of miR-580-5p on cell proliferation was investigated in HCC. Transfection with miR-580-5p inhibitor significantly decreased the expression of miR-580-5p in Huh-7 and HepG2 cells (Figure 4D). The CCK-8 assay indicated that the miR-580-5p inhibitor also inhibited the cell proliferation rate (Figure 4E), which was confirmed with EdU staining assay to detect DNA synthesis levels (Figure 4F and 4G). The cell cycle assay showed fewer cells in the S phase after miR-580-5p inhibitor transfection (Figure 4H). These results indicated for the first time that miR-580-5p might function as an oncogene in the development of HCC.

### miR-580-5p up-regulate the CCL2 expression by decrease the expression of PPARα

**Figure 5:**
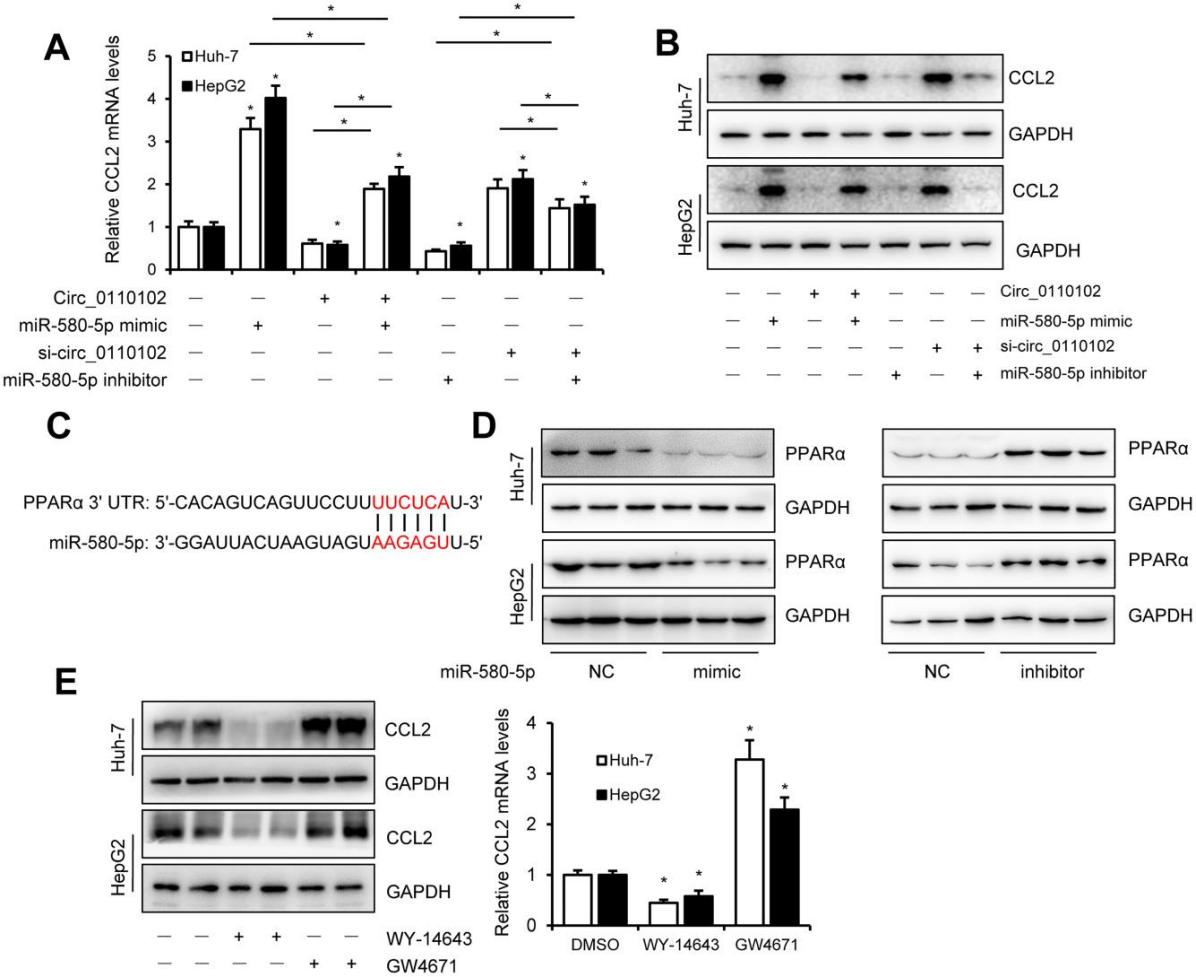
**miR-580-5p up-regulate the CCL2 expression by decrease the expression of PPARα.** (**A** and **B**) Huh-7 and HepG2 cells were co-transfected with miR-580-5p mimic or inhibitor and hsa_circ-0110102 expression vector or vector control for 48 h. CCL2 mRNA and protein levels were detected with RT-qPCR and Western blot analysis. (**C**) Predicted binding sites of miR-580-5p in the 3′ UTR of PPARα. (**D**) Huh-7 and HepG2 cells were transfected with miR-580-5p mimic or inhibitor for 48 h, the protein levels of PPARα were detected with western blot assay. (**E**) Huh-7 and HepG2 cells were treated with 5 μM WY-14643 or 10 μM GW4671 for 48 h, the protein and mRNA levels of CCL2 were detected with western blot and RT-qPCR. Data are presented as mean ± standard error. *, *P* < 0.05.

miR-580-5p mimic or inhibitor and hsa_circ_0110102 expression vector or siRNA were used to examine whether hsa_circ_0110102 and miR-580-5p effect the expression of CCL2 in HCC cells. The results showed that miR-580-5p mimic significantly increased the CCL2 mRNA and protein levels, while inhibitor decreased the level, overexpression hsa_circ_0110102 abolished the miR-580-5p mimic and si-hsa_circ_0110102 abolish the miR-580-5p inhibitor-induced downregulation of mRNA and protein expression levels of CCL2 (Figure 5A and 5B). The overexpression of miR-580-5p increased the migration and invasion of Huh-7 cells, and hsa_circ_0110102 significantly inhibited the effect of miR-580-5p (Supplementary Figure 3). Previous studies have found that, PPARα activators were able to inhibit the secretion of CCL2 [19], and the expression of PPARα was noticeably decreased in HCC tissues [20], more interestingly, a search on Targetscan (http://www.targetscan.org/) revealed the existence of a binding site between miR-580-5p and the 3′-UTR of PPARα (Figure 5C). The miR-580-5p mimic decreased, and miR-580-5p inhibitor increased the protein levels of PPARα in both Huh-7 and HepG2 cells (Figure 5D), and GW4671, the antagonist or WY-14643, the agonist of PPARα could block or increase the CCL2 protein and mRNA levels in Huh-7 and HepG2 cells (Figure 5E). These results indicating that PPARα activator was able to inhibit the expression of CCL2, miRNA-580-5p negatively regulated the PPARα level, and hsa_circ_0110102 functioned as a sponge for miR-580-5p to effect the expression of CCL2 through the activation of PPARα in HCC cells.

### AS1842856 inhibited the CCL2-induced expression of CCR2 and cytokine secretion in macrophages

**Figure 6:**
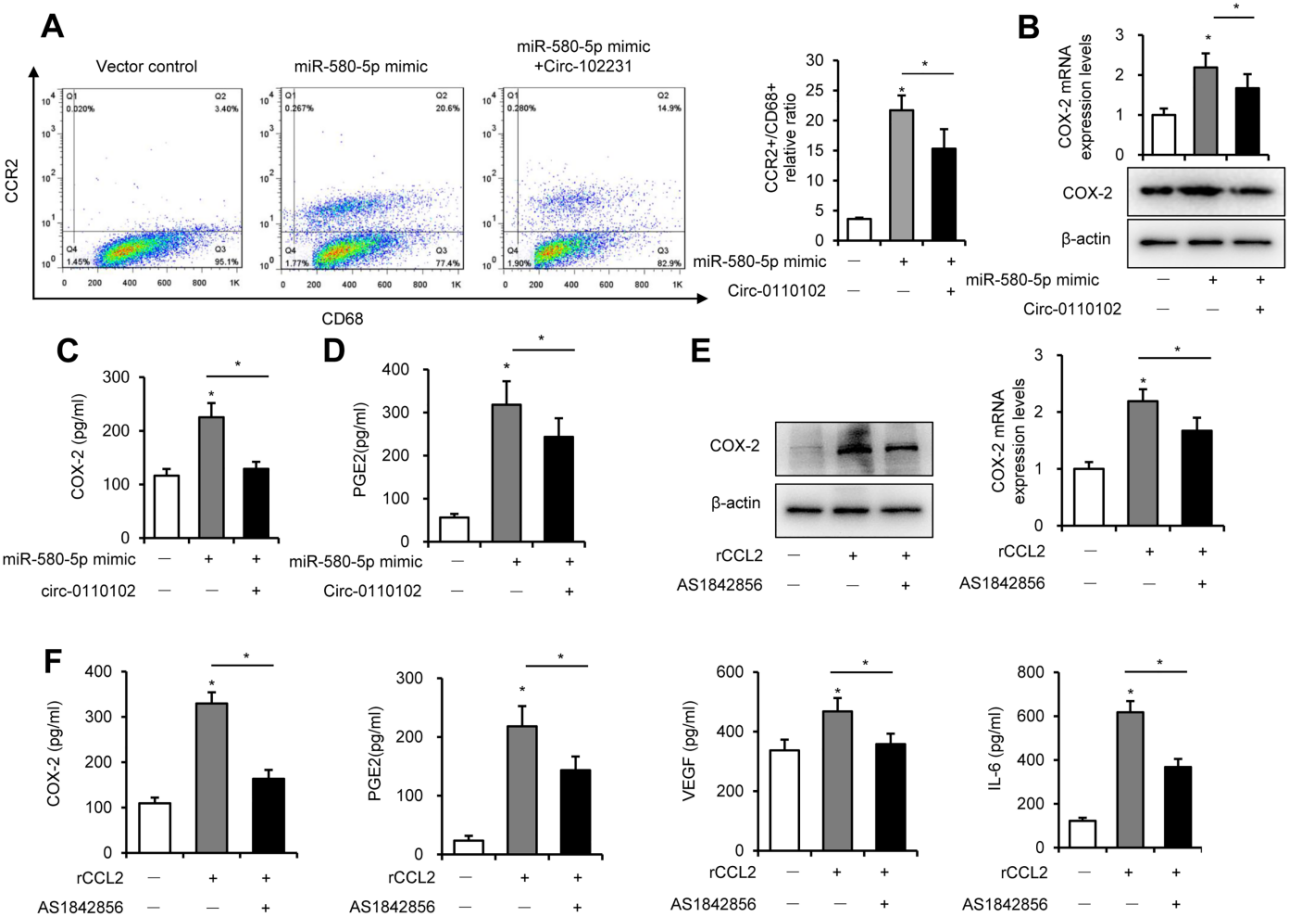
**CCL2-induced expression of COX-2 via FoxO1. Huh-7 cells were transfected with miR-580-5p mimic or hsa_circ_0110102, and then co-cultured with 200 ng/mL PMA pretreated THP-1 cells for 24 h.** (**A**) THP-1 cells were incubated with CD68 and CCR2 antibodies, followed by flow cytometry to analyze the expression of CCR2. (**B**) CCL2 protein and mRNA levels in THP-1 cells were detected by RT-qPCR and Western blot analysis. (**C** and **D**) The levels of CCL2 and PGE2 in the supernatant of the medium were measured using ELISA. (**E** and **F**) THP-1 cells were treated with 50 ng/mL rCCL2 and 1 μM AS1842856 for 48 h. (**E**) The COX-2 protein and mRNA levels were detected with RT-qPCR and Western blot analysis. (**F**) The levels of COX-2, PGE2, VEGF, and IL-6 in the supernatant of the medium were measured using ELISA. Data are presented as mean ± standard error. ^*^, *P* < 0.05.

Previous studies found that CCL2 secreted from hepatocytes triggered macrophage recruitment and induced liver fibrosis and even HCC [21]. In the tumor microenvironment, CCL2 interacted with CCR2 on the surface of macrophages to mediate the chemotaxis of monocytes and TAM to facilitate cancer progression [22]. The results showed that hsa_circ_0110102 acted as a sponge for miR-580-5p and inhibited the expression and secretion of CCL2 from HCC cells. Next, the THP-1 and Huh-7 co-culture system was used to detect their effect on the macrophage phenotype and function. The FCM results showed that CD68^+^ macrophages developed polarized CCR2 phenotypes in THP-1. Also, the percentage of CCR2^+^CD68^+^ cells increased significantly in the miR-580-5p mimic group, but co-transfection with hsa_circ_0110102 blocked the effect of miR-580-5p (Figure 6A).

Previous studies showed that COX-2 and PGE2 in the tumor microenvironment, released from macrophages, were critical in the HCC development. As shown in Figure 6B and 6C, miR-580-5p significantly induced the mRNA and protein expression of COX-2 in THP-1 cells and increased the content of COX-2 in the co-culture medium. COX-2 is a rate-limiting enzyme in regulating PGE2 generation, and its expression is highly inducible in macrophages [23]. PGE2 induced the secretion of VEGF to accelerate neovascularization in HCC [24]. Moreover, miR-580-5p induced and hsa_circ_0110102 blocked the expression of PGE2 in the co-culture system (Figure 6D).

Previous studies found that FoxO1 promoted pro-inflammatory cytokine release from macrophages [25]. The activation of FoxO1 was also involved in the regulation of the expression of COX-2 [26]. Therefore, it was suspected whether an increase in CCL2-induced expression of COX-2 in macrophages was mediated in a FoxO1-dependent manner. As shown in Figure 6E, 6F and Supplementary Figure 4, 50 ng/mL rCCL2 significantly upregulated the mRNA and protein levels of COX-2, PGE2, VEGF, and IL-6 into the medium. Either FoxO1 knockdown or inhibitor AS1842856 significantly inhibited the effect of rCCL2, instead of blocking it. These results showed that CCL2 induced the COX-2/PGE2 pathway partly through FoxO1.

### CCL2 induced FoxO1 activation via the p38 MAPK pathway in THP-1 cells

**Figure 7:**
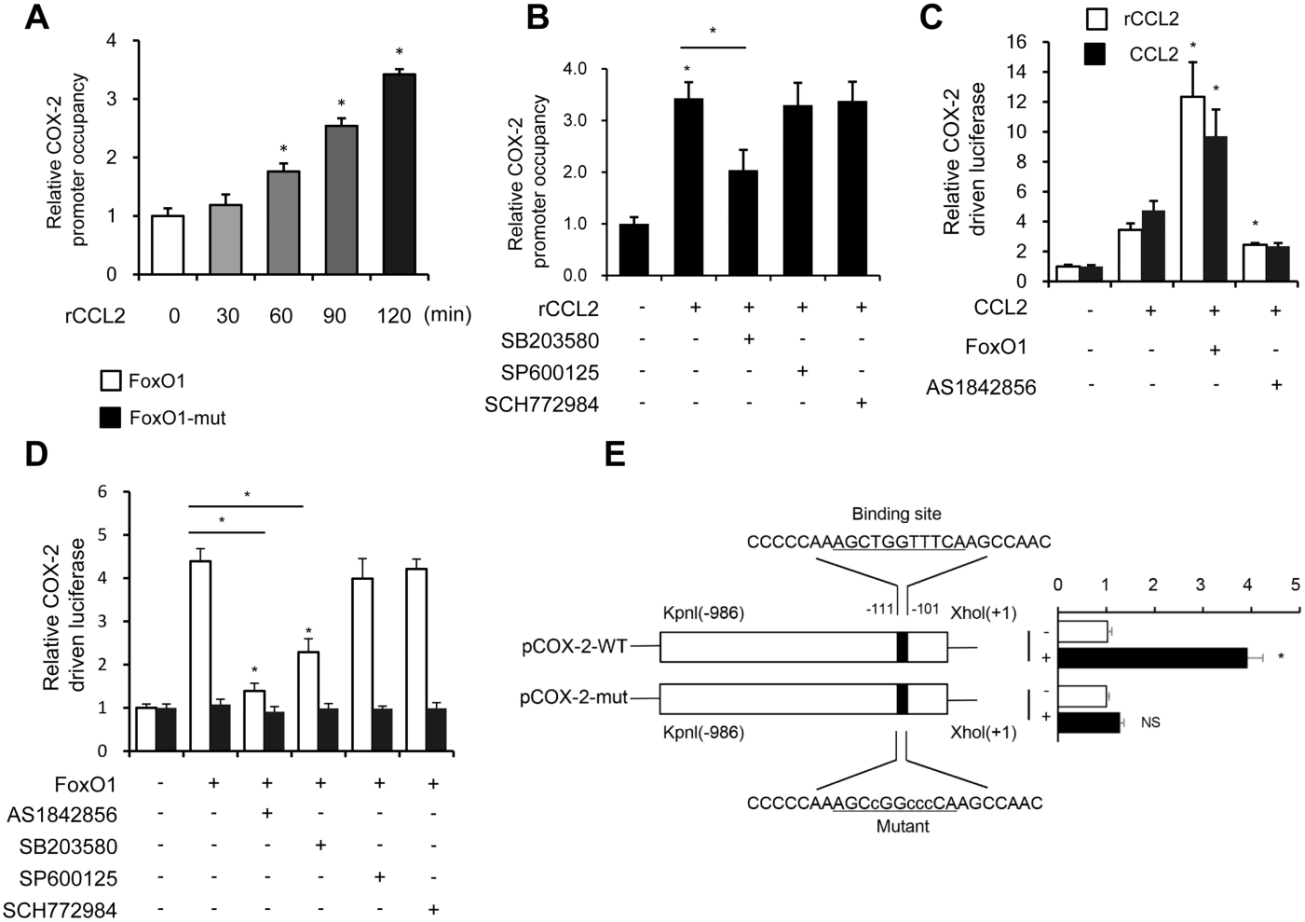
**CCL2 induced FoxO1 activation in a p38 MAPK-dependent manner**. (**A** and **B**) THP-1 was incubated with rCCL2 (50 ng/mL) for the indicated time intervals or pretreated with 10 μM SB203580, 10 μM SP600125, or 50 nM SCH772984 for 24 h, and then incubated with rCCL2 (50 ng/mL) for 120 min. The ChIP assay was applied to examine the enrichment of FoxO1 onto the COX-2 promoter. (**C** and **D**) THP-1 cells were transfected with FoxO1 expression vectors (50 ng), and pGL3-COX-2 (COX-2 promoter-driven luciferase) construct (200 ng) plus pRL-TK Renilla (10 ng). After 36 h, the cells were treated with or without AS1842856 (1 μM) or plus 10 μM SB203580, 10 μM SP600125, or 50 nM SCH772984 for a further 24 h. (**E**) THP-1 cells were co-transfected with a wild-type COX-2 promoter reporter gene (*Luc-COX-2-WT*) or FoxO1 binding site-mutated COX-2 promoter reporter gene (*Luc-COX-2-mut*) with FoxO1. The COX-2 promoter activity was determined. Luciferase activity was normalized with Renilla luciferase values. Data are expressed as fold change relative to the level of control. Data are presented as mean ± standard error. ^*^, *P* < 0.05.

Previous studies found that the MAPK pathway activated the FoxO family [27]. Interestingly, autocrine CCL2 was associated with the activation of the MAPK pathway [28]. Next, the relationship between the MAPK pathway and FoxO1 activity in response to rCCL2 stimulation in the macrophage was investigated. The ChIP assay found that rCCL2 stimulation significantly promoted the binding of FoxO1 on COX-2 promoter in a time-dependent manner in THP-1 cells (Figure 7A), which was reversed by p38 inhibitor SB203580, while the JNK inhibitor SP600125 and ERK1/2 inhibitor SCH772984 showed no effect on the COX-2 promoter activity (Figure 7B). These results demonstrated that CCL2 induced FoxO1 activation via p38 MAPK.

Both rCCL2 and forced overexpression of CCL2 were found to increase COX-2 promoter transcription activity using luciferase assay. FoxO1 combined with CCL2 caused a more significant increase, while pretreatment with AS1842856 inhibited the transcription level (Figure 7C). FoxO1-Δ256, lacking the transactivation domain of FoxO1, was used to detect whether this site was involved in regulating COX-2 activity. THP-1 cells were co-transfected with FoxO1 wild type and Δ256 expression vectors via luciferase assay. WT was found to enhance COX-2 transcription activity, but no similar enhancement was observed in Δ256 mut. Treatment with AS1842856 and p38 MAPK inhibitors both decreased the COX-2 transcription activity, but ERK and JNK inhibitors showed no effect (Figure 7D). This implied that in the physiological state, the Δ256 site of FoxO1 was quite important for the transcript activation effect of COX-2.

Next, a COX-2 promoter upstream region mutation was subcloned into pGL3-basic to define the FoxO1 target site on the COX-2 promoter. After co-transfection with FoxO1 into THP-1 cells, the activity of promoter variants was determined. As shown in Figure 7E, the activity of promoter variants with DNA deletions up to –111 nt and –101 nt showed no difference in the presence or absence of FoxO1, but FoxO1 significantly induced the WT promoter activity. These results indicated that the FoxO1 target site was localized to the –111/–101-nt region of the COX-2 promoter.

## DISCUSSION

CircRNAs are endogenous noncoding RNAs regarded as potential novel diagnostic and prognostic molecular biomarkers in cancer [29]. Recently, mounting evidence identified the important roles of circRNAs in the carcinogenesis and development of HCC, but its functional role in regulating the crosstalk between cancer cells and microenvironment was rarely explored. The microenvironment composed of fibroblasts, endothelial cells, and immune system cells, which interacted with tumor mass, could promote the progression of cancer [30]. Among these, macrophages accounted for a large proportion in tumors, and were also called TAMs. Notably, TAM acted directly on tumor cells and indirectly on the tumor microenvironment through cytokines, such as VEGF and IL-6, enhancing cancer cell invasion and metastasis, as well as angiogenesis [31].

Macrophages participate in the inflammatory environment of mutagenesis in the early stage of tumor development, and as the tumor progresses to malignancy, it stimulates angiogenesis and promotes tumor migration, invasion, and inhibition of tumor immunity [32]. Many clinical studies have shown that macrophages may promote tumorigenesis. CircRNAs have also shown a potential effect on the tumor microenvironment; circRNA CDR1as might be vital in immune and stromal cell infiltration in tumor tissue, especially those of CD8^+^ T cells, activated NK cells, and M2 macrophages [33]. circRNA circASAP1 could mediate TAM infiltration by regulating the miR-326/miR-532-5p-CSF-1 pathway [9]. Therefore, targeting circRNA to the tumor microenvironment might be a potential direction in the future.

This study focused on hsa_circ_0110102 to discover novel HCC therapy targets and found that hsa_circ_0110102 was downregulated in HCC tissues and the knockdown of hsa_circ_0110102 inhibited cell proliferation, invasion, and migration *in vitro*. As circRNAs might act as a sponge for miRNA to regulate the target gene function, luciferase reporter assays and bioinformatic prediction revealed that hsa_circ_0110102 might compete for binding with miR-580-5p.

Aberrant expression of miR-580-5p in many tumors, such as glioma and breast cancer [34], has been investigated. But until now, its exact mechanistic contribution to HCC progression has not been explored. This study indicated that miR-580-5p levels in tumor tissues were dramatically higher than that in the adjacent normal tissue, and miR-580-5p could promote the proliferation, invasion, and migration of HCC. This study further confirmed that hsa_circ_0110102 acted as an endogenous sponge on miR-580-5p. miR-580-5p decreased the expression of PPARα, which could regulate the expression of CCL2. CCL2, as a chemokine, binds to CCR2 and is chemotactic for monocytes and macrophages. It is an important human CC chemokine showing a strong tendency to monocytes. It is crucial in physiopathological reactions such as inflammation, pathogen infection, and tumor formation [35]. Many studies have shown that CCL2 is closely related to tumorigenesis and is related to poor prognosis in various cancers such as HCC [36], lung cancer, and prostate cancer [37]. Blocking the CCL2–CCR2 signaling pathway inhibits malignant tumor growth and metastasis, reduces postoperative recurrence, and improves survival [38].

A growing number of cellular signaling pathways have been implicated in orchestrating the process of COX-2/PGE2 pathway in tumor development. COX-2 acts as a rate-limiting enzyme of PGE2, is considered to be an important mediator in inflammatory responses, and is highly expressed in a variety of tumors. It interacts with many cytokines and other members of the PGE family to constitute a complex network system. Several studies suggested that high levels of PGE2 were also involved in the inflammatory response, predominant in promoting tumor growth, and associated with poor prognosis. Moreover, *in vivo* studies further indicated that the specific inhibition of COX-2 had a protective effect on HCC development and slowed down tumor progression in mouse models [39].

FoxO1, an important transcription factor, is involved in various cellular functions and activated in response to a wide range of external stimuli such as growth factors, insulin, and oxidative stress [40]. Recently, FoxO1 activation was shown to participate in COX-2/PGE2-dependent cell migration [41]. *In vitro* assays were performed to testify the effect of transcription factor FoxO1 on CCL2, inducing COX-2/PGE2 pathway activation, to comprehensively understand the effect of CCL2 on macrophages. The results showed that FoxO1 knockdown and inhibition decreased the expression of COX-2 and PGE2 and inhibited the migration and tube formation. The ChIP assay was used to investigate the binding effect of FoxO1 on COX-2 promoter, and the luciferase assay was performed to analyze the inhibitory effect of CCL2 on COX-2 promoter transcription activity so to further explore the role of the FoxO1 pathway in regulating the COX-2/PGE2 pathway in macrophages. FoxO1 WT was used to activate the endogenous activity, and Δ256 mut-FoxO1, a loss-of-function FoxO1 mutation, was used as a negative control. As expected, FoxO1 increased the COX-2 transcription activity, but Δ256 mut-FoxO1 showed no similar effect. Further, the binding site of FoxO1 on the COX-2 promoter using a point mutation promoter luciferase vector was detected.

**Figure 8:**
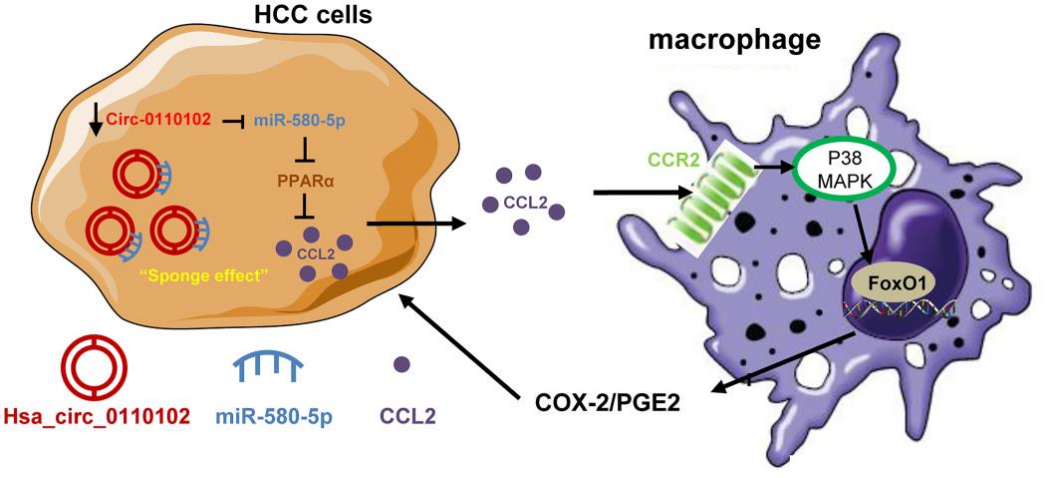
**The proposed model illustrated the role of hsa_circ_0110102/miR-580-5p/PPARα/CCL2 signaling in regulating HCC metastasis.** hsa_circ_0110102 was downregulated in patients with HCC and metastasis or recurrence and bound to miR-580-5p, which targeted PPARα 3′ UTR to down-regulate its expression level, and increased the synthesis and secretion of CCL2 from HCC cells to tumor microenvironment. CCL2 activate the COX-2/PGE2 pathway in macrophage via FoxO1 in a p38 MAPK-dependent manner, which increased the proliferation and invasion of HCC cells.

In conclusion, the findings of this study indicated a critical regulatory role of hsa_circ_0110102 as an oncogenic circRNA through the sponge effect of miR-580-5p to inhibit the expression of CCL2 in HCC cells. CCL2 could further activate the COX-2/PGE2 pathway in macrophage in a FoxO1-dependent manner (Figure 8). Overall, the present study showed that targeting the hsa_circ_00110102/miR-580-5p/CCL2 axis might be a promising therapeutic strategy for HCC.

## Supplementary Materials

Supplementary Figures
