# Supplementary material for: CircRNA hsa_circ_0110102 inhibited macrophage activation and hepatocellular carcinoma progression via miR-580-5p/PPARα/CCL2 pathway
Source: Aging (Albany NY). 2021 Apr 23;13(8):11969–87. doi: 10.18632/aging.202900 (PMC8109088; doi:10.18632/aging.202900)
Supplement: Supplementary Figures [file aging-13-202900-s001.pdf]

SUPPLEMENTARY FIGURES

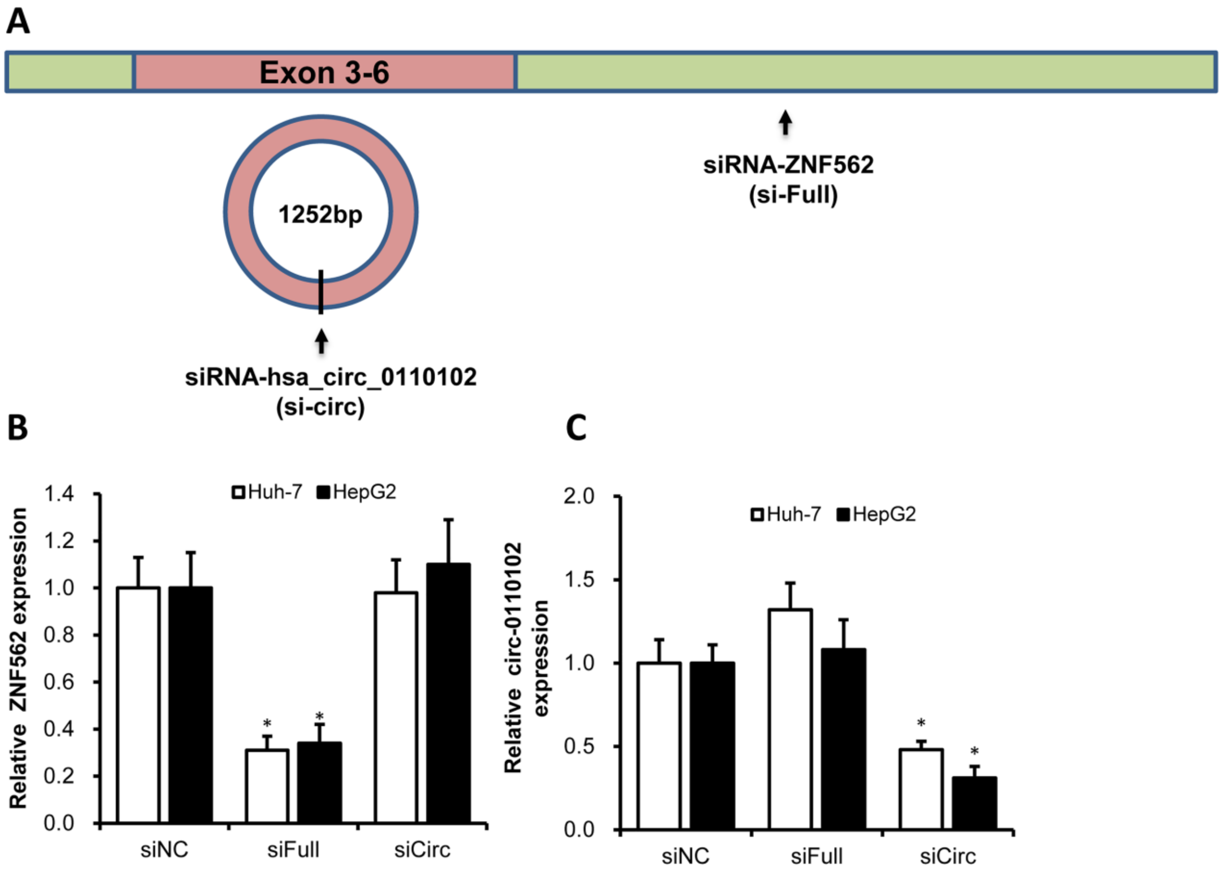

Supplementary Figure 1. hsa_circ_0110102-specific siRNA down-regulated the expression levels of hsa_circ_0110102 but not ZNF562. (**A**) Schematic model of the siRNAs. siFull (siRNA of ZNF562) targets the ZNF562 linear transcript, siCirc (siRNA of hsa_circ_0110102) targets the back-splice junction of hsa_circ_0110102. (**B**–**C**) Real-time PCR analysis of ZNF562 and circ-0110102 expression in HCC tumor cell lines Huh-7 and HepG2 with siFull or siCirc transfection. Data are shown as mean ± S.E., *n* = 3, $^{*}P < 0.05$.

<table>
<tr><th colspan="6">TargetScan miRNA predictions</th></tr>
<tr><th>CircRNA Mirbase ID</th><th>CircRNA (Top) – miRNA (Bottom) pairing</th><th>Site Type</th><th>CircRNA Start</th><th>CircRNA End</th><th>context+ score percentile</th></tr>
<tr><td>hsa_circ_0110102 (5' ... 3')
hsa-miR-338-3p (3' ... 5')</td><td>CUCUACAGAGAUGUGAUGCUGGA
| | | | | | |
GUUGUUUUAGUGACUACGACCU</td><td>8mer-1a</td><td>183</td><td>190</td><td>97</td></tr>
<tr><td>hsa_circ_0110102 (5' ... 3')
hsa-miR-766 (3' ... 5')</td><td>CUACAGAGAUGUGAUGCUGGAGA
| | | | | | | | | | |
CGACUCCGACACCCCGACCUCA</td><td>8mer-1a</td><td>185</td><td>192</td><td>99</td></tr>
<tr><td>hsa_circ_0110102 (5' ... 3')
hsa-miR-659 (3' ... 5')</td><td>GGGAAAUACAACCUAGAACCAAA
| | | | | | |
ACCCCUGGGAGGGACUUGGUUC</td><td>8mer-1a</td><td>259</td><td>266</td><td>98</td></tr>
<tr><td>hsa_circ_0110102 (5' ... 3')
hsa-miR-580-5p (3' ... 5')</td><td>GCUGUGCAUCUUGAAAUUCUCAA
| | | | | | |
GGAUUACUAAGUAGUAAGAGUU</td><td>8mer-1a</td><td>564</td><td>571</td><td>96</td></tr>
<tr><td>hsa_circ_0110102 (5' ... 3')
hsa-miR-595 (3' ... 5')</td><td>CCAUCACAACUUCCUCACACUUA
| | | | | | | | | | |
UCUGUGUGGUGCC-GUGUGAAG</td><td>8mer-1a</td><td>709</td><td>716</td><td>98</td></tr>
<tr><td>hsa_circ_0110102 (5' ... 3')
hsa-miR-188-3p (3' ... 5')</td><td>UAUGAAUGUGUUGAAUGUGGGAA
| | | | | | |
ACGUUUGGGACGUACACCCUC</td><td>8mer-1a</td><td>1188</td><td>1195</td><td>99</td></tr>
<tr><td>hsa_circ_0110102 (5' ... 3')
hsa-miR-607 (3' ... 5')</td><td>CCCAUCGUAGUAAACAUUUGAAA
| | | | | | |
CAAUAUCUAGACCUAAACUUG</td><td>8mer-1a</td><td>1228</td><td>1235</td><td>98</td></tr>
</table>

Supplementary Figure 2. A schematic model showing the putative binding sites for seven miRNAs and hsa_circ_0110102.

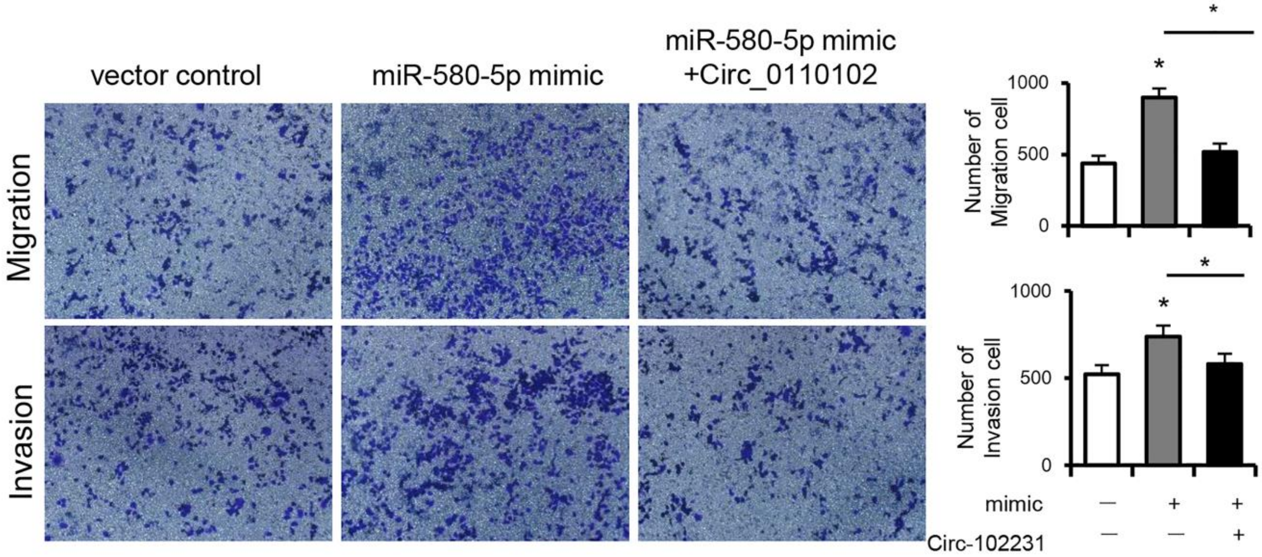

Supplementary Figure 3. The overexpression of miR-580-5p increased the migration and invasion of Huh-7 cells, and hsa_circ_0110102 significantly inhibited the effect of miR-580-5p.

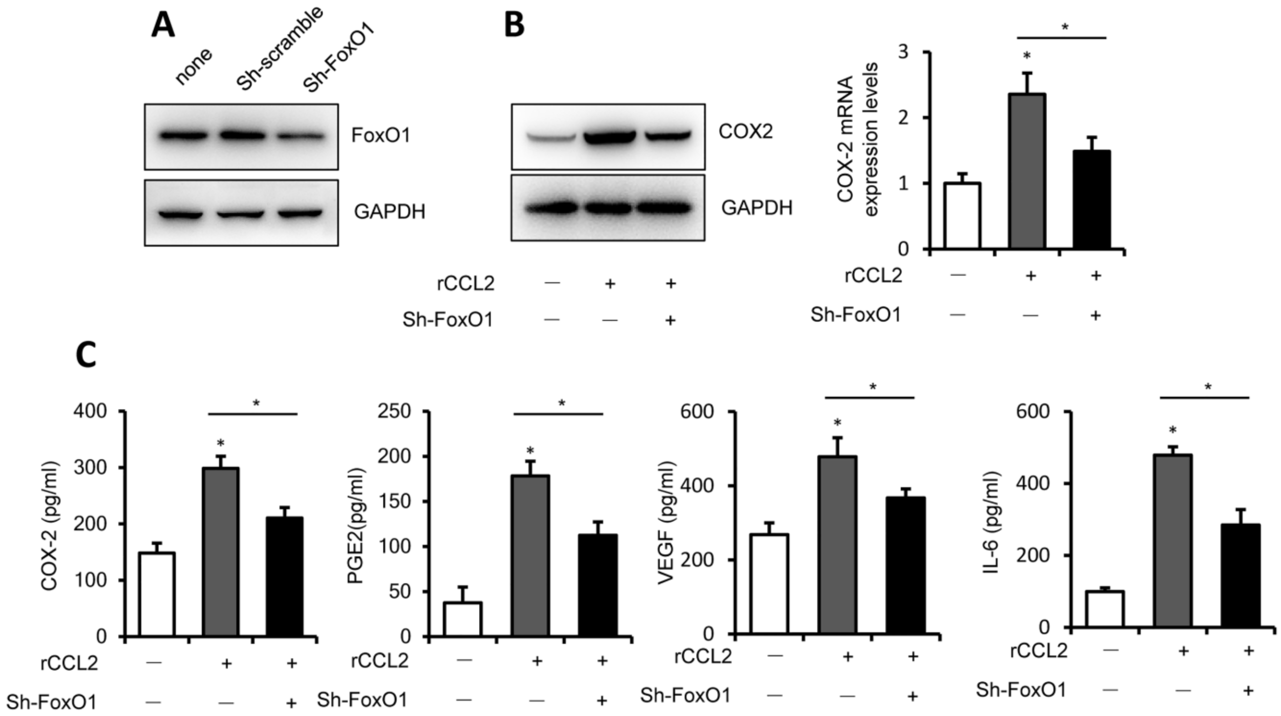

Supplementary Figure 4. FoxO1 knockdown decreased the rCCL2 induced secretion of cytokines. (**A**) sh-scramble or sh-FoxO1 were transfected into THP-1 cells for 48 h, then cells were harvested for Western blot assay. THP-1 cells were transfected with sh-FoxO1 for 24 h, and then treated with 50 ng/ml rCCL2 for another 24 h. (**B**) The COX-2 protein and mRNA levels were detected with western blot and RT-qPCR. (**C**) The contents of COX-2, PGE2, VEGF and IL-6 in the medium were measured using ELISA. Data are shown as mean ± S.E., n = 3, $^{*}P < 0.05$.
